# Codelivery of Peptide Amphiphile Nanofiber Hydrogel with Autologous Cell Suspension Facilitating Full-thickness Skin Regeneration

**DOI:** 10.1093/jbcr/irag033.577

**Published:** 2026-04-06

**Authors:** Fiona Melanie Wood, Tristan Clemons, Beatriz Da Silva Campos, Andrew Stevenson

**Affiliations:** Burns Service of Western Australia, Perth, WA; University of Southern Mississippi, Hattiesburg, MS; University of Western Australia, Perth, WA; University of Western Australia, Perth, WA

## Abstract

**Background/Objective:**

Full thickness burns cause extensive tissue loss creating a wound bed with impaired vascularization often requiring a staged surgical approach utilizing a dermal matrix to improve wound bed readiness for skin grafting. Introduction of a second stage procedure increases the time required for definitive closure. To address this, we engineered a sprayable peptide amphiphile (PA) nanofiber hydrogel functionalized with an angiogenic peptide sequence designed to be co-delivered with skin cell suspension autograft (SCSA). This bioactive supramolecular polymer scaffold preserves cell viability during high-shear spray delivery while providing the necessary chemical and mechanical environment, highly mimetic of the lost extracellular matrix, to promote vascularized tissue regeneration following injury.

**Methods:**

Electron microscopy, rheology and thixotropic recovery tests was used to confirm PA nanofiber formation and to assess scaffold porosity following spray delivery. Cytocompatibility and scaffold infiltration were analyzed using LDH release, Calcein-AM staining, and confocal microscopy with human embryonic kidney cells. Angiogenesis was quantified with an in vitro human umbilical vein endothelial cell tube formation assay. Translational relevance used a porcine full-thickness excision model, where skin cell suspension autograft delivered with PA scaffolds by spray application.

**Results:**

Hydrogels exhibited shear-thinning behavior and complete modulus recovery post-spray. No cytotoxicity was observed in the presence of the PA nanofibers, and the scaffold supported rapid cell infiltration following spray delivery with HEK293 cells. Incorporation of an angiogenic peptide sequence to the PA nanofibers significantly enhanced angiogenic metrics including nodes, junctions, and segment length (*p*<.05) in the HUVEC tube forming assay. Finally, in vivo assessment in porcine wounds treated with these bioactive nanofiber scaffolds plus autologous cells healed comparably to SCSA treated wounds demonstrating potential earlier vascularization and improved tissue organization. Flow cytometry and OCT imaging further supports the capacity for regenerative healing.

**Conclusions:**

Sprayable, bioactive PA nanofiber hydrogels enable co-delivery of viable autologous cells and scaffolds in one minimally invasive step. This approach unites material adaptability, self-healing nanostructures, and pro-angiogenic signaling to enhance burn wound healing potential while maintaining compatibility with established clinical workflows.

**Applicability of Research to Practice:**

This platform can be deployed at time of surgery to extend the clinical impact of skin cell suspension autografts. By improving cell localization, reducing donor site burden, and ensuring uniform coverage of irregular wounds, sprayable PA nanofiber scaffolds could shift practice from wound coverage to true functional regeneration.

**Funding for the study:**

We acknowledge funding support from the National Institutes of Health that supported this work.

